# Supplementary material for: Assessing spatiotemporal variability in SARS-CoV-2 infection risk for hospital workers using routinely-collected data
Source: PLoS One. 2023 Apr 21;18(4):e0284512. doi: 10.1371/journal.pone.0284512 (PMC10121006; doi:10.1371/journal.pone.0284512)
Supplement: Attachment — Submitted filename: PlosOne review_COVID.docx [file pone.0284512.s003.docx]

PlosOne review

**General comments**

1. Congratulations to authors for this very important study that sought to describe the risk of SARS-CoV-2 among health care workers in terms of spatiotemporal variability.
2. Well conducted study with sound methodology
3. Though limited by the retrospective nature of the data, authors used well the data for the study

**Specific comments**

1. A few typographical errors noted and authors should proofread thoroughly before resubmission eg line 90. Check the spelling of **storey**
2. As the availability and use of PPEs happens to be a major factor in infection spread, authors should let readers know how healthcare workers use PPE and follow the safety protocols. Would have expected others to include that in the model as an important confounder. Authors can also reference if any studies of the use of PPE.
3. A comment on the presence or otherwise of vaccines during the second wave would also enhance the study.
4. A documentation of the kind of patient contact would also be key. Are they seeing general patients or patients with SARS-COV-2? Will the analysis of that dichotomy enhance the study?
