# Supplementary material for: Assessing spatiotemporal variability in SARS-CoV-2 infection risk for hospital workers using routinely-collected data
Source: PLoS One. 2023 Apr 21;18(4):e0284512. doi: 10.1371/journal.pone.0284512 (PMC10121006; doi:10.1371/journal.pone.0284512)
Supplement: Attachment — Submitted filename: Response to Reviewers.docx [file pone.0284512.s004.docx]

Response to reviewers

We would like to thank the editor and two reviewers for taking the time to read and comment on our manuscript. Reference to line numbers are for the revised manuscript with tracked changes. Our response is in bold.

ACADEMIC EDITOR

The study describes the association between healthcare workers COVID-19 test results and behavioral markers derived from routinely collected hospital data. Congratulations on a detailed and generally well-written paper. The reviewers have provided useful comments to consider before the paper can be accepted for publication. In addition, please pay attention to the typographical errors that exist in the manuscript e.g. line 70 (environment), line 72 (variations).

**Thank you for the kind words. We have addressed any typographic errors that we have identified.**

Reviewer #1

I'd like to thank for asking me to review this interesting paper which aims to estimate the degree to which different factors affect the probability of HCWs testing positive for COVID-19 during each of the three identified stages of the pandemic and to demonstrate the epidemiological relevance of deriving markers of staff behaviour from electronic records.

The authors should consider these published papers to deep discuss their results:

doi: 10.3390/ijerph182413053

doi:10.1001/jamanetworkopen.2021.15699

doi: 10.3390/vaccines10122058

**Thank you for these additional references, we have incorporated the first two into the manuscript.**

Reviewer #2

General comments

1. Congratulations to authors for this very important study that sought to describe the risk of SARS-CoV-2 among health care workers in terms of spatiotemporal variability.
2. Well conducted study with sound methodology
3. Though limited by the retrospective nature of the data, authors used well the data for the study

**Thank you to the reviewer for the kind comments and for taking the time to review our manuscript.**

Specific comments

1. A few typographical errors noted and authors should proofread thoroughly before resubmission eg line 90. Check the spelling of storey

**We have now proof read the manuscript and made changes to any identified typographic errors.**

2. As the availability and use of PPEs happens to be a major factor in infection spread, authors should let readers know how healthcare workers use PPE and follow the safety protocols. Would have expected others to include that in the model as an important confounder. Authors can also reference if any studies of the use of PPE.

**We have added a sentence in the discussion (L392) to say ‘Future studies should consider expanding our DAG and, where possible, include data on variables that were not observed in this investigation e.g. PPE use and supply.’**

3. A comment on the presence or otherwise of vaccines during the second wave would also enhance the study.

**We detail in the methods (L102) that a ‘mass-vaccination programme began (December 8th 2020)’. We have added a brief sentence in the discussion (L388) to acknowledge the potential bias created from the vaccinations.**

4. A documentation of the kind of patient contact would also be key. Are they seeing general patients or patients with SARS-COV-2? Will the analysis of that dichotomy enhance the study?

**We agree that the type of patient contact is important. In our analysis we included models to investigate the effect of patient contacts in the context of COVID-19 patients (see methods and results). More nuanced details of the interaction type (e.g. aerosol generating procedure, lines drains and airways etc.) would also be worth investigation as these are in the routine collected data. However, due to the need for further data processing, such an analysis was beyond the scope of the one presented here.**
